# Case report: pheochromocytoma presenting as acute ST-segment elevation myocardial infarction without coronary artery abnormalities

**DOI:** 10.3389/fendo.2026.1868268

**Published:** 2026-08-12

**Authors:** Jingyuan Yi, Qianfeng Xiao, Zhihua Zhao

**Affiliations:** 1 General Inpatient Department, West China Health Care Hospital, Sichuan University, Sichuan, China; 2 Department of Cardiology, West China Hospital, Sichuan University, Sichuan, China; 3 Physical Examination Center, Wang Jiang Hospital, Sichuan University, Sichuan, China

**Keywords:** adrenal tumor, blood pressure variability, hypertension, myocardial infarction, myocarditis, pheochromocytoma, Takotsubo syndrome

## Abstract

Pheochromocytoma can cause various cardiovascular complications, but presenting as acute ST-segment elevation myocardial infarction (STEMI) without coronary artery abnormalities is rare. We report the case of a young patient to highlight the associated diagnostic challenges. A 26-year-old female presented with acute chest pain, ST-segment elevation, and elevated cardiac enzymes. Coronary angiography revealed no stenosis. She developed severe heart failure with blood pressure variability (ranging from 153/120 mmHg to 60/40 mmHg). Laboratory results showed elevated catecholamine levels, and imaging identified a 9.7×7.6 cm adrenal mass. Histopathology confirmed pheochromocytoma. This case suggests that pheochromocytoma should be considered in young patients presenting with STEMI and heart failure without coronary lesions, particularly when accompanied by blood pressure variability. It offers a systematic differential diagnosis framework for distinguishing pheochromocytoma crisis from Takotsubo syndrome and acute myocarditis in emergency settings.

## Highlights

- Key Clinical Message: Pheochromocytoma can mimic STEMI in young patients; blood pressure variability is a critical diagnostic clue.
- Why This Case Is Important: Provides a systematic differential diagnosis framework (**Table 1**) addressing the diagnostic gap in young patients with apparent acute coronary syndrome.
- How It Affects Clinical Practice: Offers actionable clinical pearls for emergency and intensive care clinicians facing similar diagnostic challenges.
- Future Research Needed: Diagnostic algorithms and prospective registries for young patients with STEMI and normal coronary arteries.

**Table 1:** Differential diagnosis framework for acute chest pain with ST-segment elevation and elevated cardiac enzymes without coronary stenosis.

Core feature	Acute myocardial infarction (AMI)	Takotsubo syndrome (TTS)	Pheochromocytoma crisis	Acute myocarditis
Coronary angiography	Stenosis or spasm	Normal	Normal	Normal
Wall motion	Segmental (coronary distribution)	Classic apical ballooning	Variable, reversible	Diffuse
BNP vs troponin	Both markedly elevated; degree related to infarct size & HF	BNP >> troponin (classic dissociation)	Both elevated, may be disproportionate	Often disproportionate (BNP higher depending on HF severity)
Key clue	Coronary lesion on imaging	Emotional/physical stress trigger	BP variability + severe tachycardia	Recent viral infection history
Prognosis	Depends on reperfusion timing	Good, recovery in weeks	Good if treated promptly	Variable (fulminant to mild)

AMI, acute myocardial infarction; TTS, Takotsubo syndrome; BNP, B-type natriuretic peptide; HF, heart failure; BP, blood pressure; STEMI, ST-segment elevation myocardial infarction.

## 1 Introduction

Pheochromocytoma is a rare neuroendocrine tumor that secretes catecholamines. A global meta-analysis reported a prevalence of approximately 19.8 cases per million population and an annual incidence of 1.9 cases per million person-years (1). The classic clinical triad of headache, palpitations, and diaphoresis occurs in fewer than half of patients, while hypertension—either paroxysmal, persistent, or with marked variability—affects approximately 90% of cases (2). Arrhythmias are another common cardiovascular manifestation of pheochromocytoma, including sinus tachycardia, atrial fibrillation or flutter, supraventricular tachycardia, and ventricular tachycardia; these patients typically present with palpitations (2, 3). Catecholamines released into the bloodstream can activate myocardial β-receptors, which increases myocardial automaticity and triggers arrhythmias. While arrhythmias are a common clinical manifestation of pheochromocytoma and can be fatal in their own right, pheochromocytoma also precipitates life-threatening cardiovascular events in addition to these arrhythmias, including acute myocardial infarction (AMI), Takotsubo syndrome (TTS), myocarditis, and aortic dissection (2). Among these, presenting as ST-segment elevation myocardial infarction (STEMI) without coronary artery abnormalities is rare, with only isolated case reports documented in the literature (2, 4, 5). When patients present with typical STEMI features-chest pain, ST-segment elevation, and elevated cardiac enzymes—but with normal coronary angiography, clinicians often struggle to identify the underlying cause. Missing pheochromocytoma can lead to catastrophic consequences, as standard STEMI management (e.g., anticoagulation, thrombolysis, beta-blockers) can exacerbate the clinical condition in pheochromocytoma-associated myocardial infarction due to the risk of spontaneous coronary dissection, tumor rupture, and unopposed alpha-mediated vasoconstriction (6, 7). Distinguishing pheochromocytoma-induced myocarditis from TTS and true AMI is clinically critical, as management strategies differ significantly.

Here, we present a case of a young female who initially presented with classic STEMI and severe heart failure, yet coronary angiography revealed no abnormalities. Through careful analysis of blood pressure patterns, tachycardia, and cardiac biomarkers, we suspected pheochromocytoma crisis, confirmed by laboratory testing, imaging, and histopathology.

## 2 Case description

**Figure 1:**
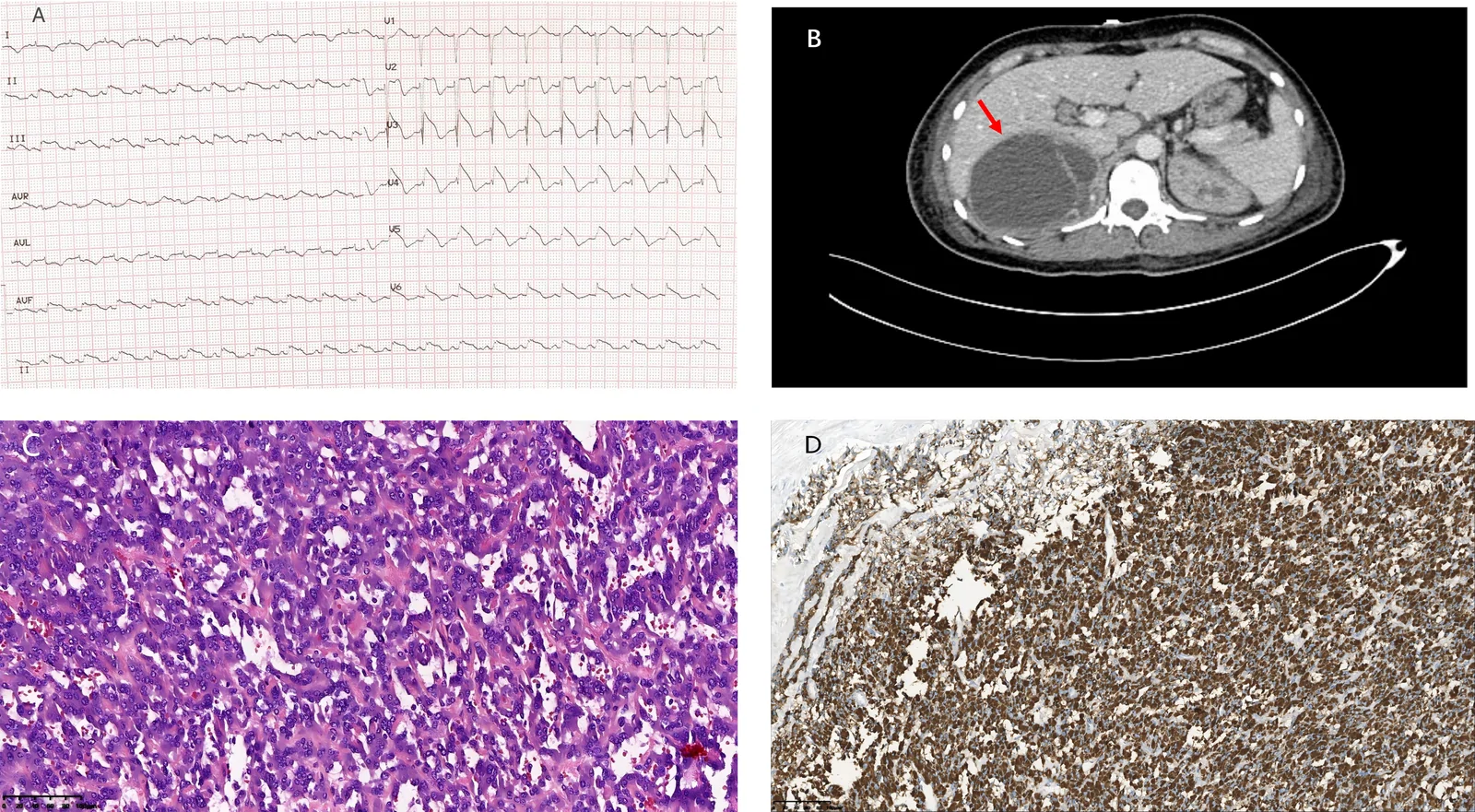
**(A)** ECG shows sinus tachycardia and ST-segment elevation in leads V1 to V6, II, III and aVF. **(B)** Abdominal CT reveals a 9.7×7.6 cm right adrenal mass (red arrow). **(C)** HE staining demonstrates cytoplasm basophilic structure with pseudo-adenoid pattern. **(D)** Positive immunohistochemical staining for Chromogranin A (CgA).

A 26-year-old female presented to the emergency department with recurrent headaches and no prior history of hypertension, cardiac disease, or recent infections. The next day in emergency department, she developed acute chest pain accompanied by palpitations. Electrocardiogram (ECG) revealed sinus tachycardia with ST-segment elevation in leads V1 to V6, II, III, and aVF (**Figure 1A**). Cardiac enzyme testing showed significant elevation of myoglobin (Myo), creatine kinase isoenzyme (CK-MB), and troponin T (TnT). Emergent coronary angiography demonstrated no coronary artery stenosis or spasm.

She was subsequently transferred to the Cardiac Care Unit (CCU). Upon admission, physical examination revealed: heart rate 142 bpm, blood pressure 153/120 mmHg, oxygen saturation 89%. There was no cardiomegaly or heart murmur, and the heart rhythm was regular, but gallops in the apical area and wet rales at the bottom of the lungs were audible upon auscultation. There was gentle tenderness in the upper abdomen, and no abdominal mass was palpable. There was no edema in the lower extremities. She developed severe dyspnea on admission to the Cardiac Care Unit (CCU). Chest X-ray demonstrated pulmonary edema. Echocardiography revealed a mild decrease in left ventricular ejection fraction (the left ventricular dimension was 48 mm and the left ventricular ejection fraction was 53%), accompanied by reduced apical wall motion and mild left atrial enlargement. Laboratory findings are summarized in **Table 2**.

Based on findings from electrocardiography, coronary angiography, cardiac biomarkers, echocardiography, and clinical presentation, a provisional diagnosis of fulminant myocarditis was established. Consequently, urapidil was administered for continuous blood pressure control throughout the course, combined with vasodilators, non-invasive ventilatory support, antibiotics, and glucocorticoids. Given the patient’s concomitant renal impairment and persistent anuria, high-dose diuretic therapy was initiated, along with intermittent continuous renal replacement therapy (CRRT) via continuous venovenous hemodialysis (CVVHD) and continuous venovenous hemodiafiltration (CVVHDF). During CCU monitoring, we observed unexplained transient hypotensive episodes, with blood pressure dropping to 60/40 mmHg. Considering the patient’s headaches alongside the observed hypotensive episodes, we hypothesized a pheochromocytoma crisis, given the triad of hypertension, headache, and hemodynamic instability. Subsequent investigations revealed markedly elevated serum norepinephrine and epinephrine levels on catecholamine testing. Computed tomography (CT) demonstrated a large right adrenal mass measuring approximately 9.7×7.6 cm (**Figure 1B**). The final diagnosis was stress-induced cardiomyopathy secondary to pheochromocytoma. Following preoperative preparation with phenoxybenzamine, the patient underwent successful right adrenal mass resection. Histopathological examination confirmed pheochromocytoma (**Figures 1C, D**).

The patient recovered well postoperatively, with blood pressure returning to normal. At the 3-month follow-up, the patient remained asymptomatic, with no evidence of recurrence.

## 3 Discussion

Pheochromocytoma is a rare neuroendocrine tumor that secretes catecholamines. In patients with hypertension, the prevalence ranges from 0.1% to 0.6% (8). Cardiovascular manifestations of pheochromocytoma are often life-threatening and may serve as the initial presentation, leading patients to seek emergency care before an endocrine etiology is identified (9). Although headache, palpitations, and diaphoresis are considered the classic triad, this triad is observed in only a minority of patients, which may contribute to diagnostic delays. Acute myocardial infarction (AMI) represents a rare complication within the spectrum of these cardiovascular emergencies. Although case reports of pheochromocytoma presenting as STEMI have been published (5, 10–12), this case contributes several unique elements: First, our patient was a 26-year-old female, while most reported cases involve older patients. Accordingly, in young patients with acute coronary syndrome, particularly those exhibiting refractory hypertension, paroxysmal palpitations, or exercise-induced nausea and vomiting (13), early measurement of 24-hour urinary catecholamines or their metabolites (such as metanephrines) and abdominal imaging are recommended to improve diagnostic accuracy and minimize delays in treatment initiation. Second, we developed **Table 1** to provide a systematic differential diagnosis framework-a practical tool for distinguishing pheochromocytoma crisis from TTS and acute myocarditis in emergency settings. Third, marked blood pressure variability, particularly unexplained paroxysmal hypertension punctuated by hypotension, is a crucial clinical indicator for pheochromocytoma, and continuous hemodynamic monitoring can facilitate early diagnosis (14). The dramatic blood pressure variability (ranging from 153/120 mmHg to 60/40 mmHg) was the pivotal clinical clue, demonstrating the diagnostic value of hemodynamic monitoring.

**Table 2:** Laboratory findings on admission.

Makers	Level	Reference
WBC	31.12*10^9	3.5-9.5*10^9
RBC	6.12*10^12	3.8-5.1*10^12
PLT	604*10^9	100-300*10^9
HCT	5.5L/L	3.5-4.5L/L
NT-proBNP	>35000pg/mL	0-153pg/mL
TnT	9900ng/L	0-14ng/L
CK-MB	140.7ng/mL	<2.88ng/mL
Myo	>3000ng/mL	<58ng/mL
ALT	86IU/L	<40IU/L
AST	314IU/L	<35IU/L
Crea	219μmol/L	37-110μmol/L

WBC, white blood cell; RBC, red blood cell; PLT, platelet; HCT, hematocrit; NT-proBNP, N-terminal pro-B-type natriuretic peptide; TnT, troponin T; CK-MB, creatine kinase-MB isoenzyme; Myo, myoglobin; ALT, alanine transaminase; AST, aspartate transaminase; Crea, creatinine.

Pheochromocytoma crisis can cause acute myocardial injury through several mechanisms: First, increased catecholamine levels trigger vasoconstriction, which reduces coronary blood flow. Concurrently, catecholamines enhance myocardial contractility and accelerate heart rate, leading to increased myocardial oxygen demand (5, 12). Second, pheochromocytoma crisis may induce hemoconcentration and hypovolemia (15), which reduce coronary blood flow and trigger myocardial ischemia. Third, massive catecholamine secretion may cause myocardial stunning and stress cardiomyopathy, namely Takotsubo syndrome (TTS), which leads to elevated myocardial enzymes and ST-segment changes. If a patient develops TTS induced by pheochromocytoma, a decrease in left ventricular ejection fraction (LVEF) may lead to thrombosis; embolic dislodgement can result in coronary artery occlusion (11). Additionally, studies have shown that massive catecholamine secretion may trigger coronary artery spasm (12). Moreover, some clinical evidence indicates that norepinephrine spillover caused by pheochromocytoma can also induce acute myocarditis and myocardial injury, leading to myocardial necrosis, inflammatory cell infiltration, and fibrosis (2, 12, 16).

Distinguishing pheochromocytoma crisis from TTS and acute myocarditis is clinically critical (**Table 1**).

As previously mentioned, pheochromocytoma crisis can induce TTS or acute myocarditis. TTS and acute myocarditis are clinically difficult to differentiate, as both may present with ECG changes, elevated myocardial enzymes, and reversible cardiac dysfunction. However, the mechanism of TTS involves catecholamine-induced myocardial stunning; myocardial enzymes are usually moderately elevated, while B-type natriuretic peptide (BNP) or N-terminal pro-brain natriuretic peptide (NT-proBNP) levels are often markedly elevated (17, 18), resulting in a mismatch between the degree of myocardial enzyme elevation and BNP/NT-proBNP elevation. In contrast, the mechanism of acute myocarditis induced by pheochromocytoma involves direct myocardial injury by catecholamines. Thus, myocardial enzymes are markedly elevated in patients with acute myocarditis, and the degree of myocardial enzyme elevation is usually consistent with that of BNP/NT-proBNP. Additionally, compared with acute myocarditis, TTS is typically characterized by apical ballooning on imaging.

This case provides actionable clinical pearls:

- Consider pheochromocytoma in young patients (<30 years) presenting with apparent STEMI and heart failure without coronary abnormalities.
- Blood pressure variability-from severe hypertension to hypotension-is a hallmark feature of pheochromocytoma crisis.
- Cardiac biomarkers (troponin, BNP/NT-proBNP) help differentiate pheochromocytoma-induced myocarditis from TTS and true AMI.
- Early recognition enables timely alpha-blockade and definitive tumor resection for favorable outcomes.

This case presents several limitations. First, myocardial biopsy and CMR were precluded due to the patient’s critical condition. Second, current clinical guidelines recommend germline genetic testing for all patients diagnosed with pheochromocytoma and paraganglioma (PPGL). This disease carries a high genetic risk; results from such testing offer critical clinical guidance for long-term patient surveillance and screening of high-risk family members (19, 20). However, genetic testing was not performed in this case. Finally, as a single case report, the findings cannot be generalized to the broader population. Nevertheless, this case still provides valuable clinical insights for the fields of emergency medicine and critical care.

Given the rarity and atypical presentation of pheochromocytoma-induced STEMI with angiographically normal coronary arteries, this condition is frequently misdiagnosed, underscoring the necessity of rigorous differential diagnosis in emergency settings. Owing to the rarity of the disease, large-scale multicenter trials remain impractical. Consequently, it is essential to establish a standardized diagnostic workflow and a dedicated prospective multicenter clinical registry targeting young patients with Myocardial Infarction with Non-Obstructive Coronary Arteries (MINOCA) and labile hypertension. Such real-world registry data can effectively elucidate distinct clinical features, determine the actual disease incidence, and optimize individualized management strategies for this often underrecognized and high-risk subgroup.

## 4 Conclusion

Pheochromocytoma should be considered in young patients presenting with acute myocardial infarction and acute heart failure without coronary artery lesions, particularly when accompanied by hypertension and blood pressure variability. This case contributes a systematic differential diagnosis framework (**Table 1**) that can guide clinicians in distinguishing pheochromocytoma crisis from Takotsubo syndrome and acute myocarditis. Early recognition enables accurate diagnosis and appropriate management, ultimately improving patient prognosis.

## Patient perspective

The patient provided written informed consent and approved the publication of this case report. After successful adrenal surgery, the patient recovered well and was placed on regular follow-up. At the 3-month follow-up, the patient remained asymptomatic, with normal blood pressure and no evidence of tumor recurrence.

## Data Availability

The original contributions presented in the study are included in the article/**Supplementary Material**. Further inquiries can be directed to the corresponding author.
