# Supplementary material for: Case report: pheochromocytoma presenting as acute ST-segment elevation myocardial infarction without coronary artery abnormalities
Source: Front Endocrinol (Lausanne). 2026 Aug 12;17:1868268. doi: 10.3389/fendo.2026.1868268 (PMC13506299; doi:10.3389/fendo.2026.1868268)

Supplementary Materials

Case Report: Pheochromocytoma Presenting as Acute ST-Segment Elevation Myocardial Infarction Without Coronary Artery Abnormalities

1. Serial Echocardiographic Findings

2. Pathology Report of Right Adrenal Pheochromocytoma

3. Serial Plasma and Urinary Catecholamine Levels During Hospitalization and Follow-up

All supplementary materials presented in this manuscript are extracted from the patient's original electronic medical records, including serial imaging studies, pathological reports, and laboratory test results collected during hospitalization and postoperative follow-up. Furthermore, no data have been modified or artificially generated.

1. Serial Echocardiographic Findings

Echocardiogram 1: June 26, 2017 (Admission)

Left Ventricle (LV)	48 mm	--
Left Atrium (LA)	36 mm	--
Aortic Root (AO)	28 mm	--
Aortic Valve (AV)	1.3 m/s	--
Ejection Fraction (EF)	53%	≥55%

Findings:

Mild left atrial enlargement

Normal thickness of the interventricular septum and left ventricular posterior wall

Reduced apical left ventricular wall motion

Mild-to-moderate tricuspid regurgitation (Vmax=3.2 m/s, PG=42 mmHg)

Estimated pulmonary artery systolic pressure: ~55 mmHg

Mild mitral regurgitation

Slight dilatation of the inferior vena cava (~21 mm) with <50% respiratory collapse.

Echocardiogram 2: July 28, 2017

Left Ventricle (LV)	46 mm	--
Left Atrium (LA)	37 mm	--
Right Ventricle (RV)	22 mm	--
Right Atrium (RA)	29 mm	--
Interventricular Septum (IVS)	10 mm	--
LVPW	8 mm	--
Ascending Aorta (AAO)	27 mm	--
Main Pulmonary Artery (MPA)	22 mm	--
Aortic Valve (AV)	1.1 m/s	--
Pulmonary Valve (PV)	1.2 m/s	--
Mitral E velocity (Emv)	0.9 m/s	--
Mitral A velocity (Amv)	0.5 m/s	--
Septal E' (Em)	9 cm/s	--
Septal A' (Am)	7 cm/s	--
E/e' ratio	10	--
End-Diastolic Diameter (EDD)	46 mm	--
End-Systolic Diameter (ESD)	30 mm	--
End-Diastolic Volume (EDV)	96 mL	--
End-Systolic Volume (ESV)	34 mL	--
Stroke Volume (SV)	62 mL	--
Ejection Fraction (EF)	65%	≥55%

Findings:

Left atrial enlargement

Mild tricuspid regurgitation (Vmax = 2.6 m/s, PG = 27
mmHg) Trivial mitral regurgitation

Echocardiogram 3: August 15, 2017

Left Ventricle (LV)	50 mm	--
Left Atrium (LA)	36 mm	--
Right Ventricle (RV)	20 mm	--
Right Atrium (RA)	30 mm	--
Interventricular Septum (IVS)	10 mm	--
LVPW	9 mm	--
Ascending Aorta (AAO)	29 mm	--
Aortic Valve (AV)	1.3 m/s	--
Pulmonary Valve (PV)	0.9 m/s	--
Mitral E velocity (Emv)	0.8 m/s	--
Mitral A velocity (Amv)	0.5 m/s	--
Septal E' (Em)	9 cm/s	--
Septal A' (Am)	5 cm/s	--
E/e' ratio	8.89	--
End-Diastolic Diameter (EDD)	50 mm	--
End-Systolic Diameter (ESD)	33 mm	--
End-Diastolic Volume (EDV)	120 mL	--
End-Systolic Volume (ESV)	46 mL	--
Stroke Volume (SV)	74 mL	--
Ejection Fraction (EF)	62%	≥55%

Findings:

Mild left heart enlargement

Mild tricuspid regurgitation (Vmax=2.7 m/s, PG=30 mmHg)

Trivial mitral regurgitation

2. Pathology Report of Right Adrenal Pheochromocytoma

2.1. Patient and Specimen Information

Institution	West China Hospital, Sichuan University
CAP Accreditation	College of American Pathologists (CAP) accredited
Pathology Number	[Redacted for de-identification]
Patient Sex / Age	Female / 26 years
Requesting Department	Urology
Requesting Physician	Dr. Zhu Yuchun
Specimen Received	February 2, 2018
Specimen Type	Surgical excision, right adrenal gland

2.2 Gross Examination (Macroscopic Findings)

Specimen description	One mass with a small amount of attached adrenal tissue
Tumor dimensions	8.0 cm x 7.0 cm x 6.0 cm
Adrenal tissue dimensions	8.0 cm x 4.5 cm x 0.3 cm
Weight	10 g
External surface (capsule)	Intact and smooth
Cortical thickness	0.1 cm
Cut surface	Cystic; filled with grayish-red and yellow content
Tissue blocks submitted	Blocks (1), (2), (3): tumor + adjacent adrenal tissue; Block (4): tumor alone
Sampling date	Morning, February 3, 2018
Sampling technician	Menglan Zhang
Recording technician	Dengchao Shi

Specimen: A right adrenal mass with a small amount of adjacent adrenal tissue attached.

2.3. Initial Pathology Report (Issued February 6, 2018)

Lesion site	Right adrenal gland
Sample type	Surgical excision
Diagnosis	Neoplasm; immunohistochemistry staining required for further classification

2.4. Final Pathology Report with Immunohistochemistry (Issued February 9, 2018)

Lesion site	Right adrenal gland
Sample type	Surgical excision
Final diagnosis	Pheochromocytoma

Final diagnosis: Pheochromocytoma of the right adrenal gland. Reporting pathologist: Dr. Zhang Wenyan

Immunohistochemistry (IHC) Staining Results

[redacted]		
Tumor cells	PCK (pan-cytokeratin)	Negative (-)
Tumor cells	CgA (chromogranin A)	Positive (+)
Tumor cells	alpha-Inhibin	Negative (-)
Tumor cells	MART-1 (Melan-A)	Negative (-)
Tumor cells	Ki-67 / MIB-1	Positive (+), < 3%
Sustentacular cells	S-100	Positive (+)

3. Serial Plasma and Urinary Catecholamine Levels
During Hospitalization and Follow-up

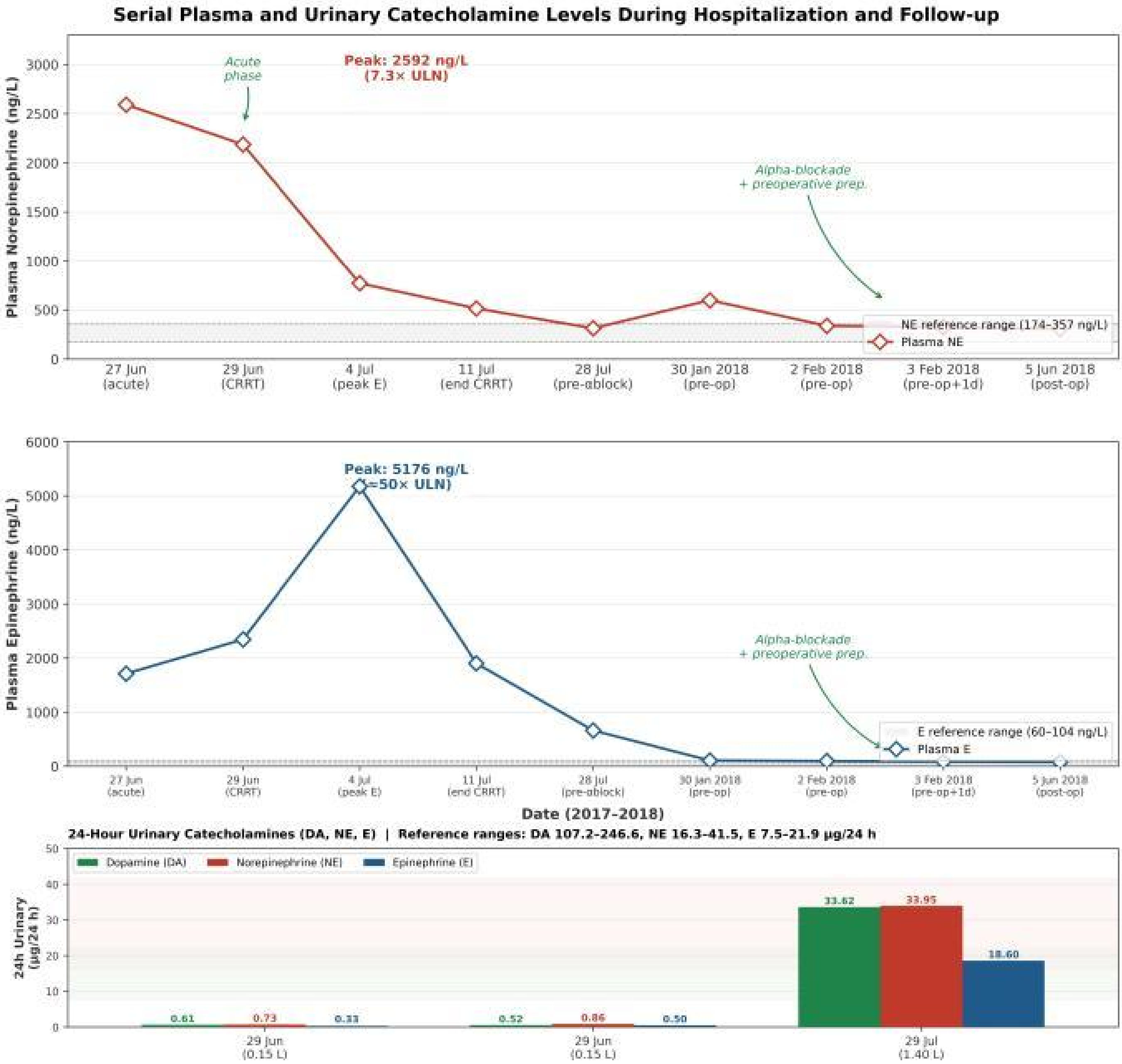

Supplement: Supplementary file 1 [file SupplementaryFile1.pdf]
